# Tirzepatide-Based Therapy for Multidomain Metabolic Improvement in an Active Duty Service Member: A Case Report

**DOI:** 10.7759/cureus.114114

**Published:** 2026-08-07

**Authors:** Shawn K Kim

**Affiliations:** 1 Internal Medicine, 51st Medical Group, Pyeongtaek, KOR

**Keywords:** active duty, case report, diabetes, masld, military, military medicine, obesity, tirzepatide, weight loss

## Abstract

Tirzepatide has demonstrated efficacy in multiple domains of metabolic health, such as weight loss, glycemic control, and improvement of metabolic dysfunction-associated steatotic liver disease (MASLD). Despite this, tirzepatide remains understudied and underutilized in the military population. We report a case of a 29-year-old active-duty male with newly diagnosed type 2 diabetes mellitus (T2DM), class II obesity (BMI 35.6 kg/m²), dyslipidemia, and MASLD who experienced marked improvement across multiple metabolic domains over approximately 12 months of tirzepatide-based treatment. This case highlights that a comprehensive treatment approach, including tirzepatide, lifestyle modifications, and adjunctive pharmacotherapy, was associated with substantial multidomain metabolic improvement in young active duty service members (ADSM). Further studies are needed to determine whether these findings translate into broader benefits for military readiness, retention, and healthcare utilization within the Military Health System (MHS).

## Introduction

Obesity, type 2 diabetes mellitus (T2DM), dyslipidemia, and metabolic dysfunction-associated steatotic liver disease (MASLD) are heavily intertwined and frequently exist together [1]. In the military population, metabolic health is especially important because active duty service members (ADSM) must maintain physical fitness, operational readiness, deployability, and retention eligibility [2]. Recent data show that about 68% of active duty service members (ADSM) are now considered overweight or obese [3], with obesity prevalence alone rising from 14.7% to 24.2% between 2013 and 2023 [4,5]. Prediabetes prevalence in the active-duty population has been estimated at close to 20%, although this figure is extrapolated rather than directly measured [6]; incidence has also risen from 588.2 to 763.2 cases per 100,000 person-years between 2018 and 2021 [7]. Because of these trends, obesity is now the number one cause of enlistment disqualification and military separation [2,3,8,9], and the Department of Defense (DoD) spends an estimated $1.25 billion annually in direct healthcare costs attributable to obesity, with the total burden exceeding $1.35 billion annually when lost productivity is included [8].

Tirzepatide, a dual glucose-dependent insulinotropic polypeptide (GIP) and glucagon-like peptide-1 (GLP-1) receptor agonist, has emerged as an effective therapy for glycemic control in patients with diabetes and chronic weight management in obesity [10-12]. Emerging evidence suggests that tirzepatide may improve hepatic inflammation and fibrosis in patients with MASLD [13]. Despite the burden of poor metabolic health and growing evidence supporting its benefits, tirzepatide appears to be both underutilized and understudied within the Military Health System (MHS) [14-16]. Only 5.5% of BMI-eligible MHS beneficiaries received an obesity medicine, a rate far below the estimated 30.5% of MHS beneficiaries who meet criteria for obesity [14].

We report a case of marked metabolic health improvement during tirzepatide-based treatment in a young ADSM with newly diagnosed T2DM, class II obesity, dyslipidemia, and MASLD.

## Case presentation

A 29-year-old active-duty male presented to urgent care for viral gastroenteritis and was incidentally found to have severe hyperglycemia. His initial laboratory evaluation revealed markedly elevated serum glucose and hemoglobin A1c (HbA1c), elevated transaminases (aspartate aminotransferase (AST) and alanine aminotransferase (ALT)) with a normal alkaline phosphatase, and severe mixed dyslipidemia with elevated total cholesterol, low-density lipoprotein (LDL) cholesterol, and triglycerides (Table 1). His weight was 108.9 kg, with a BMI of 35.6 kg/m², consistent with class II obesity. He was discharged home on insulin glargine 10 units daily with titration education.

**Table 1:** The patient's initial laboratory findings at the urgent care. Values are shown with directional flags relative to the reference range: ↑, above the reference range; ↓, below the reference range; no flag, within the reference range. Reference ranges are representative adult intervals and may vary by laboratory. AST: aspartate aminotransferase; ALT: alanine aminotransferase; GAD65: glutamic acid decarboxylase-65; HbA1c: hemoglobin A1c; IA-2: islet antigen-2; LDL: low-density lipoprotein; ZnT8: zinc transporter-8.

Laboratory tests	Baseline (Day 0)	Reference range
Glucose (mg/dL)	430 ↑	70–99 (Fasting)
HbA1c (%)	10.1 ↑	<5.7
AST (U/L)	61 ↑	0–40
ALT (U/L)	75 ↑	0–44
Alkaline phosphatase (U/L)	85	39–117
Total cholesterol (mg/dL)	307 ↑	<200
LDL cholesterol (mg/dL)	172 ↑	<100
Triglycerides (mg/dL)	456 ↑	<150
Anti-GAD65 antibodies	Negative	Negative
IA-2 autoantibodies	Negative	Negative
ZnT8 antibodies	Negative	Negative
Insulin antibodies	Negative	Negative

When he presented to his initial outpatient appointment about one week after the urgent care visit, he was using insulin glargine 14 units daily. After extensive counseling and education, multiple medication changes occurred. Tirzepatide 2.5 mg weekly was initiated, insulin glargine was reduced to 10 units daily with self-titration, and atorvastatin 20 mg daily was started. Because his autoimmune diabetes panel from the urgent care visit had tested negative (Table 1), metformin extended-release (ER) was started as well, with gradual titration. Referrals were placed to optometry and disease management for lifestyle counseling, including diet and exercise education. Right upper quadrant ultrasound (RUQUS) demonstrated diffusely increased hepatic parenchymal echogenicity with at least moderate hepatocellular disease, most consistent with hepatic steatosis. Given the absence of alcohol use and other potential etiologies of steatosis, the patient was diagnosed with MASLD.

At his one-month follow-up, he reported his overall glycemic control had improved significantly. He was instructed to increase tirzepatide to 5 mg weekly, while decreasing his insulin glargine from 10 units to six units daily and continuing metformin ER 1000 mg twice daily and atorvastatin 20 mg daily.

At the four-month follow-up, the patient endorsed tolerating tirzepatide 5 mg weekly and required insulin glargine only four units daily. Along with many lifestyle changes, his weight had decreased to 100.5 kg. Because of stable glycemic status, insulin glargine was completely discontinued and empagliflozin 10 mg daily was started. The rest of his medication regimen was continued the same, including tirzepatide 5 mg weekly.

At the six-month follow-up, the patient's HbA1c had decreased to 5.6%, and his weight was 96.4 kg. Because of continued improvement in his overall metabolic health, metformin ER was decreased to 500 mg twice daily, while continuing empagliflozin 10 mg daily and tirzepatide 5 mg weekly.

**Figure 1:**
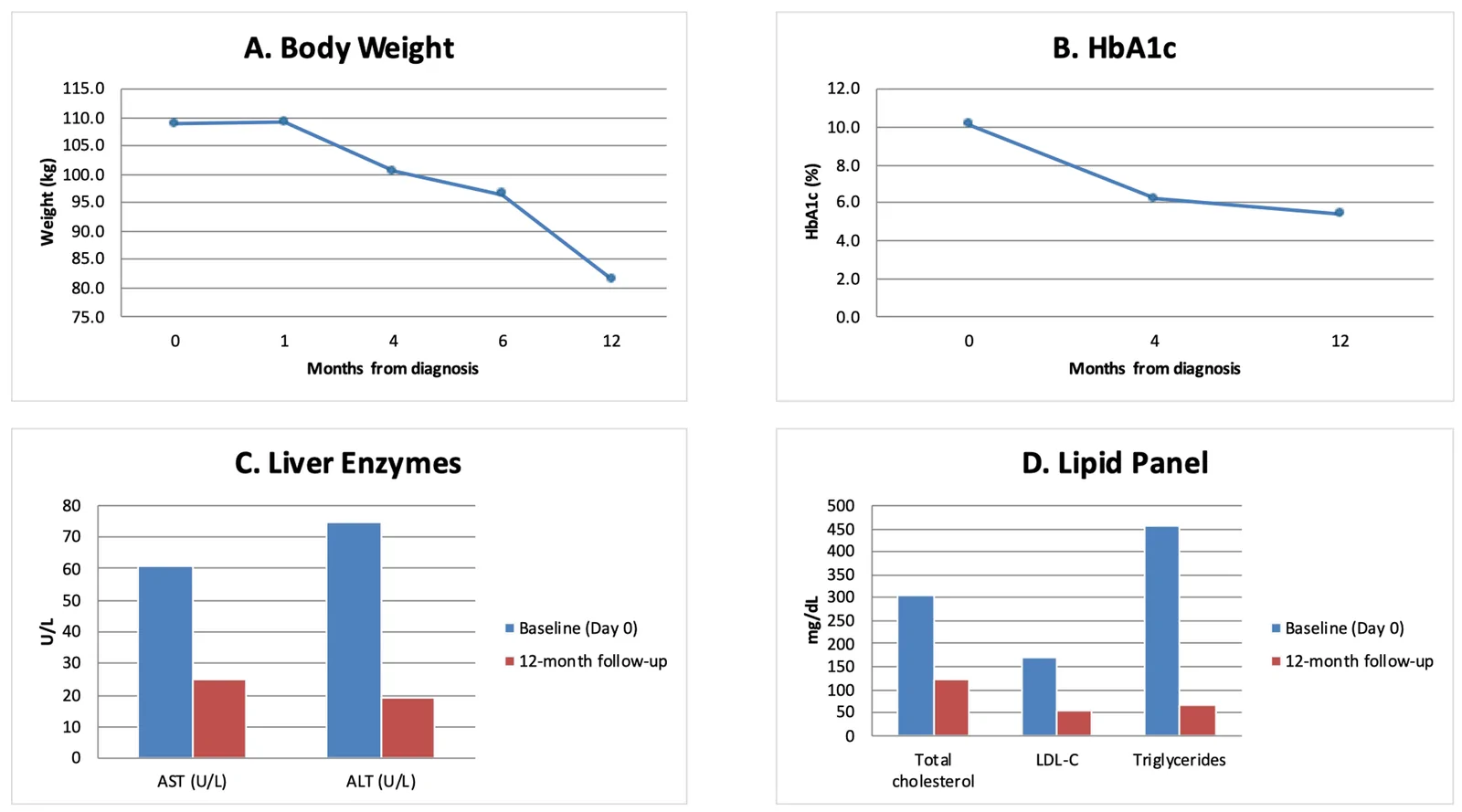
Trajectory of the patient's metabolic parameters during the tirzepatide-based treatment. (A) Body weight, (B) hemoglobin A1c (HbA1c), (C) liver enzymes (AST, ALT), and (D) lipid panel (total cholesterol, LDL-C, triglycerides) at baseline and follow-up. AST: aspartate aminotransferase; ALT: alanine aminotransferase; HbA1c: hemoglobin A1c; LDL-C: low-density lipoprotein cholesterol.

At his one-year follow-up, his repeat RUQUS showed no evidence of steatosis. The patient's weight had decreased to 81.5 kg. His transaminases improved to AST 25 U/L and ALT 19 U/L. The lipid panel improved to total cholesterol 123 mg/dL, LDL cholesterol 56 mg/dL, and triglycerides 65 mg/dL. His HbA1c later reached 5.4%. The patient was not only medically optimized but also very ecstatic about how he could confidently pass his waist measurement and physical fitness test. The trends in the patient's weight, glycemic, hepatic, and lipid parameters over the treatment course are depicted in Figure 1.

## Discussion

This case demonstrates simultaneous remissions in four domains of metabolic health: insulin-dependent T2DM, dyslipidemia, MASLD, and obesity class II (25% weight loss). These remarkable changes happened in about 12 months in a young ADSM through a tirzepatide-based treatment plan.

The progression of this patient's clinical status is closely aligned with the findings from landmark trials on tirzepatide. Adding tirzepatide to insulin glargine can reduce HbA1c by up to 2.40% and the required insulin dose [11]. Despite this patient never exceeding a 5 mg weekly dose, his weight loss (25% of baseline) exceeds the mean 20.9% reduction seen with the 15 mg dose in SURMOUNT-1 [12], likely reflecting the better efficacy of early detection and early treatment. It also emphasizes the additive contributions from metformin, empagliflozin, and lifestyle changes. His remission of MASLD, supported by the ultrasound and liver enzyme values, is consistent with histologic findings from a phase 2 trial of tirzepatide in biopsy-confirmed metabolic dysfunction-associated steatohepatitis (MASH) with fibrosis; tirzepatide allowed resolution of MASH in 44-62% of the participants compared with 10% on placebo [13].

Beyond its metabolic benefits, insulin dependence itself has operational and deployability implications for ADSM due to the requirement of daily injections, continuous glucose monitoring, and the inherent risk of severe hypoglycemia. Other aspects of poor metabolic health directly impact ADSM's retention and physical fitness for duty, as obesity and its associated comorbidities are the leading medical reasons for early military separation, lost duty time, and Military Health System cost [2,3,8,9,17]. Higher BMI is independently correlated with increased odds of various clinically diagnosed medical conditions [18], and ADSM with obesity have disproportionately higher healthcare utilization than their counterparts with normal BMI [19]. Among soldiers enrolled in the Army Body Composition Program, body fat exceeded standards by more than anticipated and 16% met criteria for metabolic syndrome, underscoring that body composition failures often reflect underlying cardiometabolic disease [20]. Nonetheless, anti-obesity medications are disproportionately underutilized among ADSM, compared with other beneficiaries in the MHS [14]. This case spotlights that aggressive and early treatment of poor metabolic health can produce readiness-relevant outcomes for the military.

This is a single case over the timespan of 12 months. Thus, it cannot establish concrete causality or generalizability. More longitudinal observation would be required to study the long-term efficacy of tirzepatide [16]. Also, the patient's positive clinical change was able to occur through intensive lifestyle changes and a multimodal regimen-tirzepatide, metformin, empagliflozin, and atorvastatin. The amount of contribution solely from tirzepatide cannot be isolated or measured. Despite these limitations, the temporal correlation between tirzepatide introduction, followed by dose escalation, and the trajectory of weight loss, glycemic control, hepatic, and lipid parameters suggests that tirzepatide, in combination with lifestyle modifications and adjunctive pharmacotherapy, was associated with the observed clinical improvements (Figure 1).

## Conclusions

This case describes substantial multidomain metabolic improvement, including insulin discontinuation for T2DM, resolution of sonographic hepatic steatosis with normalization of transaminases, normalization of lipid parameters, and 25% weight loss over approximately 12 months using a comprehensive treatment approach that included tirzepatide, metformin, a sodium-glucose cotransporter-2 (SGLT2) inhibitor, a statin, and structured lifestyle modifications. These findings suggest that tirzepatide-based therapy, as part of a multimodal treatment strategy, may be beneficial for obesity-related metabolic disease in ADSM. Further studies are needed to confirm these findings and to evaluate their potential implications for military readiness and long-term health outcomes.
